# First report of anatoxin-a producing cyanobacteria in Australia illustrates need to regularly up-date monitoring strategies in a shifting global distribution

**DOI:** 10.1038/s41598-019-46945-8

**Published:** 2019-07-26

**Authors:** Nijoy John, Louise Baker, Brendan R. E. Ansell, Steven Newham, Nicholas D. Crosbie, Aaron R. Jex

**Affiliations:** 1 Department of Veterinary Biosciences, The University of Melbourne, Parkville, Victoria 3010 Australia; 2 Population Health and Immunity Division, Walter and Eliza Hall Institute of Medical Research, Parkville, Victoria 3052 Australia; 3 Goulburn Valley Water, Shepparton, Victoria 3630 Australia; 4 Melbourne Water, Docklands, Victoria 3008 Australia; 5 School of Civil and Environmental Engineering, The University of New South Wales, Sydney, NSW 2052 Australia

**Keywords:** Water microbiology, Microbial ecology

## Abstract

Routine monitoring of toxic cyanobacteria depends on up-to-date epidemiological information about their distribution. In Australia, anatoxin producing cyanobacteria are not regularly tested for and thought to be rare if not absent from the continent. Our study investigated the presence of anatoxin-a (ATX-a) producing cyanobacteria in surface water samples (n = 226 from 67 sampling locations) collected from 2010 to 2017 across the state of Victoria, Australia. We (1) detected the presence and distribution of *ana*C (anatoxin-a synthetase C) gene sequences previously associated with various cyanobacteria, including *Cuspidothrix issatschenkoi*, *Aphanizomenon* sp., *D. circinale, Anabaena* sp., and *Oscillatoria* sp., from 31 sampling locations, and (2) determined the concentration of ATX-a in samples tested using ELISA, in two instances detected at >4 µg · L^−1^. These data present the first confirmation of ATX-a producers in Australia. Our study indicates that ATX-a should be included in regular testing of cyanobacterial blooms in Australia and highlights the importance of regular investigation of the distributions of toxic cyanobacteria worldwide, particularly amid the known expanding distribution of many cyanobacterial taxa in a period of increased eutrophication and rising surface water temperatures.

## Introduction

Cyanobacterial blooms are a major water-quality issue affecting management of surface waters globally^1^. In addition to impacting water palatability, these blooms have the potential to produce a variety of toxic secondary metabolites or “cyanotoxins”, including hepatotoxins [e.g., Microcystin (MC), Nodularin (NOD)], neurotoxins [e.g., Saxitoxin (SXT), Anatoxin (ATX)] and/or cytotoxins [e.g., Cylindrospermopsin (CYN)] of significant health, veterinary and agricultural importance^1,2^. Identification and differentiation of toxic cyanobacteria is vital to monitoring and management of various water resources, which in turn is directed by contemporary knowledge of the regional distribution of toxin types.

Many studies in the last decades, have described increasing incidence of cyanobacterial blooms and, more importantly, a rapid global expansion of cyanobacteria species into new environments^3,4^. Numerous reports indicate increased spread/prevalence of ‘invasive’ cyanobacterial species; including, for example, previously ‘tropical/subtropical’ CYN producing *Raphidiopsis raciborskii* (previously: *Cylindrospermopsis raciborskii*) and *Chrysosporum ovalisporum* (previously: *Aphanizomenon ovalisporum*) and, ATX producing *Raphidiopsis mediterranea* and *Cuspidothrix issatschenkoi* (previously: *Aphanizomenon issatschenkoi*) emerging in temperate regions of Europe, the USA, Japan and New Zealand (see reviews^5–9^).

Cyanobacterial blooms are common in many parts of Australia^10–13^ and toxic cyanobacteria capable of producing MC, NOD, CYN and SXT are widely distributed in tropical, subtropical and temperate regions of Australia^14–17^. Among various cyanotoxins produced by cyanobacteria globally, anatoxin-a (ATX-a) is a potent neurotoxic alkaloid compound that can cause severe asphyxia and muscular paralysis in humans and animals^18^. ATX-a was first isolated in the 1970’s from filamentous cyanobacteria, *Aphanizomenon flos-aquae*^19^. Since then, several ATX-a producing genera of cyanobacteria, including *Anabaena/Dolichospermum*^20–23^, *Oscillatoria*^22,24–26^, *Aphanizomenon*^22,27–29^, *Cu. issatschenkoi*^22,27–29^, *Phormidium*^30,31^ and *Tychonema*^32,33^ have been identified worldwide. While previous reports have confirmed the expansion of CYN producing *R. raciborskii* to many parts of Australia (see reviews^6–9^), and more recently *Ch. ovalisporum* to south-eastern parts of Australia^34^, understanding of the prevalence and ecology of ATX-a producers in Australia is limited. Although previous studies^10–13^ have detected small quantities of *Cu. issatschenkoi* in the Murray Darling Basin, Australia, an ATX-a producing cyanobacterium is yet to be confirmed^35^ and therefore, this toxin is not routinely tested for and presumed to be absent in Australian waters.

A range of diagnostic tools are currently used to monitor ATX-a producing cyanobacterial blooms^36^. These techniques include microscopic identification and enumeration of the phytoplankton community^37,38^, direct detection/quantification of ATX-a using Enzyme Linked Immunosorbent Assay (ELISA) or liquid chromatography with tandem mass spectrometry (LC-MS/MS)^39^, and PCR-based assays targeting specific genes in the ATX-a synthetase gene cluster^22,25,40,41^, mainly *ana*F^28^ and *ana*C^22^. More recently, a study by Legrand *et al*.^42^ has described a nested PCR-based method that is both sensitive and specific for amplifying *ana*C genes from environmental samples.

Given the availability of diagnostic tools, but lack of prevalence data for ATX-a producers, with the expansion of several cyanobacteria species (see reviews^5–9^) into new geographical regions and increasing incidence of toxic blooms, we undertook a study to investigate the emergence of ATX-a producing cyanobacteria in 226 cyanobacterial bloom samples collected over a period of seven years from various water bodies across the state of Victoria (VIC), Australia. This study combined the following methods: analyses of cyanobacteria community composition using microscopy, a nested PCR-based amplification and sequencing of environmental *ana*C genes using two existing general *ana*C primer pairs^22^ and direct detection of ATX-a by ELISA. Our study confirms the presence of ATX-a producing cyanobacteria in Australia. Moreover, this study highlights the clear need to better understand and closely monitor the rapidly shifting distributions of toxic cyanobacteria species in global surface waters and to regularly update the testing regimes for these microorganisms.

## Results

### Sample collection

**Figure 1:**
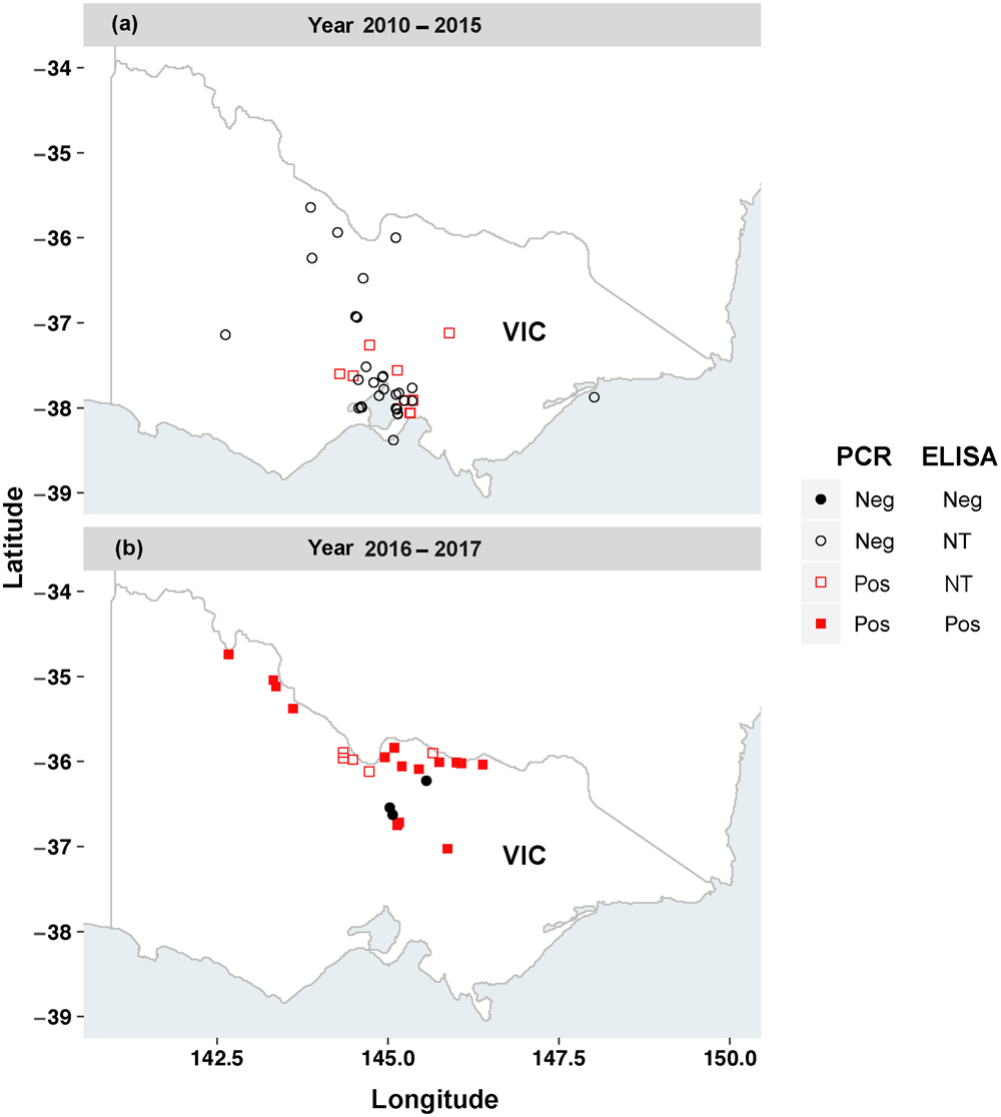
Locations of samples (**a**) Year 2010–2015, **(b**) Year 2016–2017 tested in this study for the presence of anatoxin-a (ATX-a) using nested PCR and ELISA. Points in the graph represent summation of nested PCR and ELISA data at each location tested rather than individual samples. Symbols; VIC, Victoria; Pos, Positive; Neg, Negative; NT, Not tested.

For the current study, a total of 226 surface water samples (1L surface grabs) were collected from 67 locations across the state of VIC, Australia over summer and autumn months (November to April) from 2010 until 2017 (Fig. 1). All samples were taken during bloom events and exceeded ~1000 cyanobacterial cells · mL^−1^. In some instances, samples exceeded a biovolume of 10 mm^3^ L^−1^.

### *ana*C gene nested PCR

**Figure 2:**
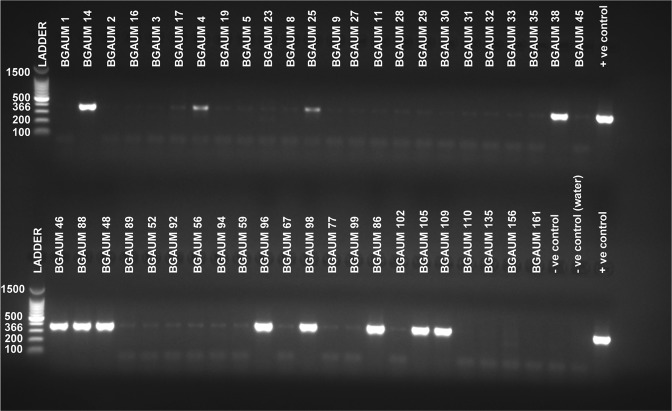
Nested PCR results of anatoxin-a synthetase C (*ana*C) gene of a subset of cyanobacterial bloom samples separated by 1.5% agarose gel electrophoresis. DNA fragments at 366 bp indicate the *ana*C gene. The positive control represents a known ATX-a producing *Cuspidothrix issatschenkoi* CY1941. A known negative ATX-a producer, *Nostoc punctiforme* PCC73102 and non-template control (water), were used as negative controls. The ladder represents DNA fragments with 100 bp. For sample descriptions and sequencing results, see Supplementary Table S1. The present image is cropped and a full-length gel is presented in Supplementary Fig. S1.

Amplification of the cyanobacterial 16S rRNA and internal spike control assays prior to *ana*C nested PCR were found to be positive for all samples. For *ana*C gene screening, DNA extracts from each of the 226 samples were tested by nested PCR using published *ana*C general primer pairs (Table 1)^22^ known to specifically target ATX-a producing cyanobacteria. In the primary PCR process, no PCR products except for the positive control were observed on a 1.5% agarose gel electrophoresis, indicating low prevalence of *ana*C target gene in our samples. Subsequent nested PCR step produced a total of 68 amplicons (from 31 sampling locations across the state of VIC, Australia) from 226 samples with an expected 366 bp size on a 1.5% agarose gel electrophoresis unit (Fig. 2). Out of the total 68 amplicons, 20 amplicons were observed as very strong bands on the agarose gel electrophoresis. The remaining amplicons, i.e., 4/68 and 44/68 were observed as strong and faint bands, respectively (see Supplementary Table S1). The majority of the amplicons (i.e., 59/68, 86.7%) in this study were generated from 82 samples, corresponding to a recent (2016/2017) major cyanobacterial bloom event spreading across 23 different locations along the Murray River and associated waterways (including rivers, creeks, lakes, canals and reservoirs) bordering the states of New South Wales (NSW) and VIC, Australia (Fig. 1b). The remaining nine *ana*C (i.e., 9/68, 13.2%) positive amplicons produced faint bands on agarose gel electrophoresis, and were represented by non-Murray bloom samples (i.e., n = 144) collected prior to 2016 (Fig. 1a).

**Table 1:** List of PCR primers^22^ used in this study to amplify anatoxin-a synthetase C (*ana*C) gene.

Primers	Sequences (5′ - 3′)	Target gene	Product size
anxgenF anxgenR	ATGGTCAGAGGTTTTACAAG CGACTCTTAATCATGCGATC	*ana*C	861 bp
*ana*C-genF *ana*C-genR	TCTGGTATTCAGTCCCCTCTAT CCCAATAGCCTGTCATCAA	*ana*C	366 bp

To identify potential ATX-a producing cyanobacteria within this recent bloom, cyanobacteria community abundance was determined for each sample by observation under light microscope. The majority of these samples contained *Ch. ovalisporum* (Forti) E. Zapomelová, O. Skácelová, P. Pumann, R. Kopp & E. Janecek 2012 (previously *A. ovalisporum*), a known CYN producer, as the dominant cyanobacterium along with *Planktolyngbya*, *Cyanogranis*, *Chroococcales* and *Aphanocapsa* as the subdominant species. Small quantities of potential ATX-a producers including *Cu. issatschenkoi* (Usaĉev) Rajaniemi *et al*. 2005 (previously: *Ap. issatschenkoi*), species belonging to Aphanizomenonaceae family and *Dolichospermum* sp., were also present (see Supplementary Table S1).

### *ana*C gene sequencing and phylogenetic analysis

To confirm the identity of *ana*C nested PCR products, the 24 very strong and strong amplicons were sequenced by bi-directional (capillary) sequencing. Twenty three amplicons produced high quality (>97%) unambiguous sequence data (analysed using Geneious® Version 10) and the remaining amplicon was removed from further analysis. A BLASTn search of the NCBI non-redundant database of each of the 23 sequenced amplicons showed 90–100% nucleotide identity to several previously annotated cyanobacteria *ana*C genes from uncultured cyanobacteria (Accession Numbers: KX096813, KT246302, KP036898), *Oscillatoria* sp. (Accession Numbers: JF803653, JF803652), *Cu. issatschenkoi* (Accession Numbers: KM245023, KM245024, KM245025), *Dolichospermum circinale* (previously: *Anabaena circinalis*) (Accession Number: JF803647), *Anabaena* sp. 37 (Accession Number: JF803645), *Anabaena* sp. 54 (Accession Number: JF803646) and *Aphanizomenon* sp. 3 (Accession Number: JF803655) (Table S1).

**Figure 3:**
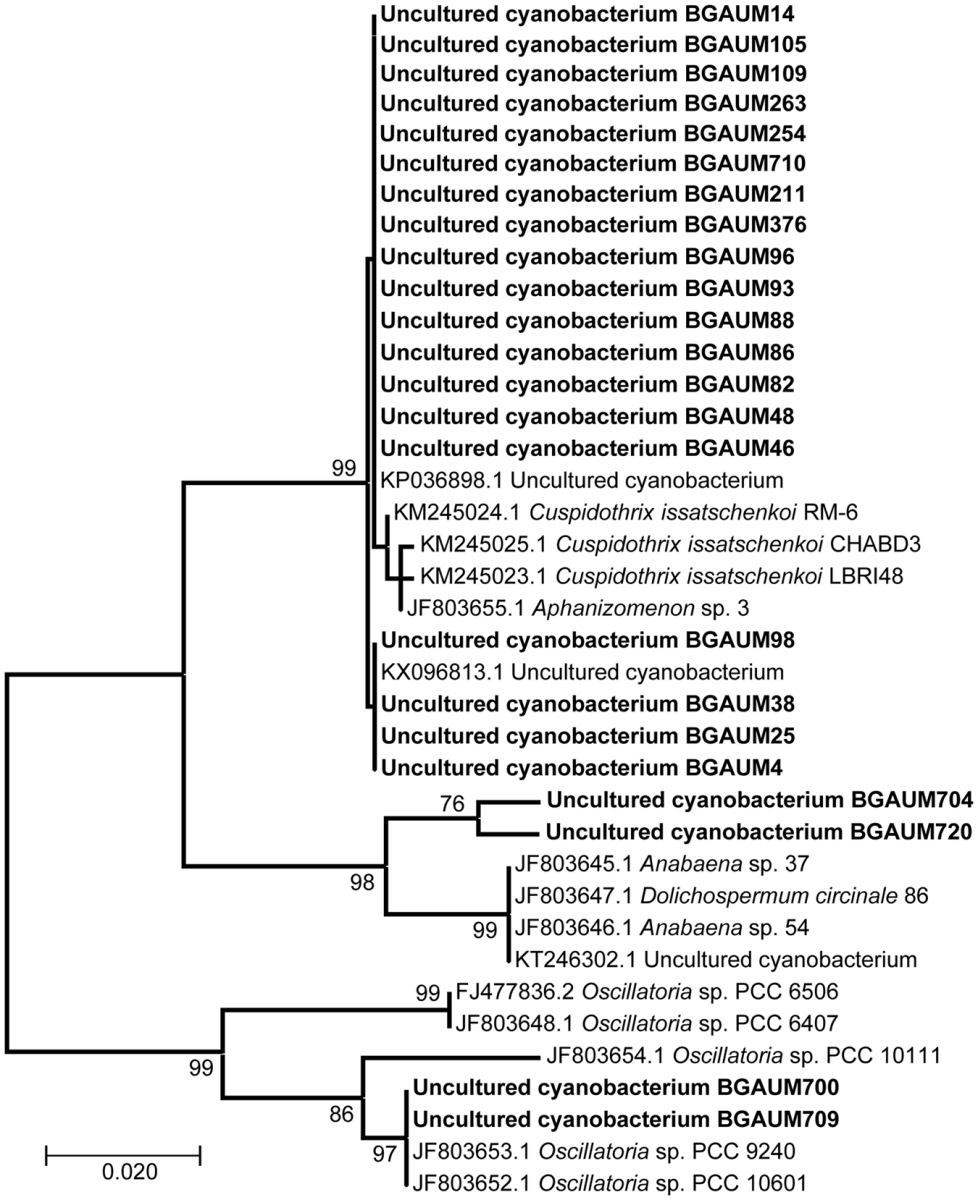
Unrooted phylogenetic tree of partial anatoxin-a synthetase C (*ana*C) gene sequences (340 bp). 23 sequences generated in this study (marked in bold) along with reference sequences from NCBI GenBank were used to create the tree. Phylogenetic analyses were conducted in MEGA7^57^ using the Maximum Likelihood (ML) based on the Tamura-Nei model^56^. Bootstrap values from 1000 replicates are shown at the nodes. The scale bar represents 0.02 substitutions/site.

All 23 *ana*C sequences obtained from this study along with other peer reviewed *ana*C sequences from GenBank were used to construct a 340 bp Maximum Likelihood (ML) tree (Fig. 3). A previously described ATX-a producing *Phormidium autumnale* CAWBG618 (Accession Number: KX016036) with a smaller *ana*C gene fragment (308 bp) was excluded from the phylogenetic analyses. The overall topology of the *ana*C gene ML tree recognizes three distinct groups with >98% bootstrap support. The majority of the sequences (19/23) derived from the current study were identical and clustered with a larger group of *ana*C sequences of many labelled in GenBank as “Uncultured cyanobacterium”, *Cu. issatschenkoi* and *Aphanizomenon* sp. The remaining four *ana*C sequences from this study clustered with two different smaller groups including several *ana*C GenBank sequences of *Anabaena* sp., *D. circinale* and *Oscillatoria* sp. (see Fig. 3 for Accession Numbers). Small genotypic variations in *ana*C genes among different ATX producing cyanobacteria have resulted in the formation of subgroups within the three major groups.

### ATX-a concentration in cyanobacterial bloom samples

**Figure 4:**
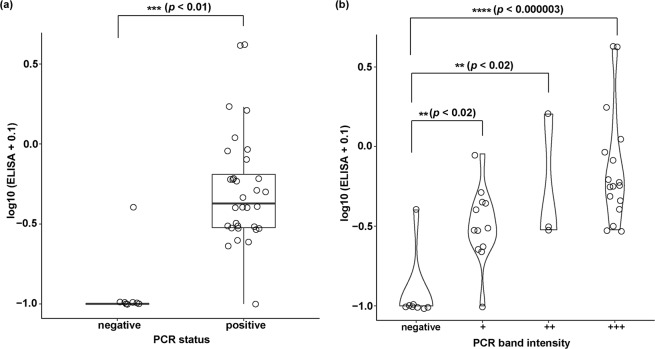
Box plot (**a**) and violin plot (**b)** representing log-transformed anatoxin-a (µg · L^−1^) measured by ELISA (Y-axis) corresponding to nested PCR results (X-axis) of 40 cyanobacterial bloom samples. The grey line across the box (A) indicates the median anatoxin-a concentration. Asteriks (*) represent statistical significance (*p* < 0.05). PCR band intentisities (B) were indicated by faint (+), strong (++) and very strong (+++) bands.

To confirm the presence of anatoxin producers in these water samples, we undertook direct ATX-a detection in 40 (32 *ana*C nested PCR positive and 8 negative) samples corresponding to a large recent (2016–2017) cyanobacterial bloom extending over 500 kms across the Murray River and associated waterways, Australia. All samples were analysed in duplicate using the Abraxis^®^ ELISA kit. A total of 29 of 32 nested PCR positive samples showed ATX-a concentrations between 0.15–1.6 µg · L^−1^, with two samples exceeding >4 µg · L^−1^ and one sample showing <0.15 µg · L^−1^. Of the eight negative nested PCR samples, seven samples showed zero or <0.15 µg · L^−1^ (the minimum reading for the test) of ATX-a and one sample had 0.3 µg · L^−1^ of ATX-a (Table S1). A significant difference (*p* < 0.01, Welch Two sample *t*-test) was observed between ATX-a ELISA concentrations of nested PCR-positive and negative samples (Fig. 4a). Additionally, the relationship between observed nested PCR band intensities (i.e., very strong, strong, weak and negative) and ATX-a ELISA values was also found to be highly significant (*p* < 0.000003 and *p* < 0.02, Tukey’s honest significant difference (HSD) test and ANOVA) (Fig. 4b). All PCR positive groups showed greater ELISA signal than the PCR negative group, and the highest PCR band intensity (‘very strong’) was greater than the lowest non-zero (‘weak’) intensity (Fig. 4b).

## Discussion

We examined the presence of ATX-a producing cyanobacteria in 226 samples from 67 locations (Fig. 1) across the state of VIC, Australia by microscopy, nested PCR and ELISA assays. Based on the microscopy data, we detected several potential/known ATX-a producing cyanobacteria in many samples (Table S1). Nested PCR testing of these same samples detected the presence of *ana*C, a gene required for ATX-a synthesis, at overall sample prevalence of 30.1% (68/226 samples tested) representing 46.2% of sample locations (31/67 sample sites: Figs 1, 2 and Table S1).

Bi-directional sequencing confirmed the identity of the nested PCR amplicons from our samples as the *ana*C gene. The phylogenetic clustering by unrooted ML analysis clustered these sequences into three distinct groups, of which the majority of ana*C* sequences most likely represent ATX-a producing *C. issatschenkoi* along with *Aphanizomenon* sp., *Oscillatoria* sp., *Anabaena* sp. and *D. circinale* (Table S1 and Fig. 3). Overall, our findings (corroborated by microscopy, nested PCR, sequencing and phylogenetic analysis) agree with previous reports of common ATX-a producing cyanobacteria in many parts of Europe^20,28,33,43,44^, Asia^41,45,46^ and New Zealand^27,30,31^.

Direct toxin detection (using the Abraxis^®^ anatoxin-a ELISA kit) confirmed the presence of ATX-a in all but one of the *ana*C positive samples (Fig. 4 and Table S1). It is not clear whether these samples represent significant health risks as currently there are no guideline values documented for ATX-a toxin and its congeners in Australia and/or by World Health Organisation (WHO). None of our samples showed toxin levels exceeding the New Zealand provisional maximum value for ATX-a in drinking water (i.e., 6 µg · L^−1^)^47^. Two exceeded a provisional guideline value (i.e., 3 µg · L^−1^) for ATX-a in drinking water outlined by the province of Oregon, USA^47^, however it should be noted that these samples were taken from raw water (i.e. pre-treatment) and not product water (post-treatment). ATX-a ELISA assay was performed on recently collected (2016–2017) samples (i.e., from the Murray River and associated waterways) only. All samples from 2010–2015 were tested by *ana*C nested PCR only. Positive *ana*C detection in these samples is indicative of their potential to produce ATX-a, but actual toxin production has not been confirmed.

Although the ELISA assay quantified ATX-a production in our samples, ELISA values did not strongly correlate with the total number/bio-volume of potential/known ATX-a producing cyanobacteria strains identified in our microscopy analysis (Table S1). The possible reasons for this are, (1) the ATX-a producing cells in our samples declined rapidly prior to our sampling, but the ATX-a did not, (2) despite planktonic cyanobacteria being known as the major ATX-a producers in surface waters, the primary source in some samples may be ATX-a producing benthic cyanobacteria species^48,49^, or (3) these samples contain other major species of ATX-a producing cyanobacteria that are yet to be identified. Of these possibilities, the latter seems most likely as all samples were transported under cold storage and processed immediately upon receipt and yet none contained large quantities of a known ATX-a producer on microscopic examination. Additionally, while microscopy could not clearly identify a major ATX-a producer at high abundance, associated *ana*C genes were readily detectable by nested PCR in all ATX-a positive samples, indicating against the primary toxin source being a benthic species not present in the surface water grabs. These possible explanations, however, must be evaluated through further exploration. Additionally, further studies using more accurate quantitative methods for direct toxin detection and quantification (i.e., HPLC-MS / GC-MS)^50,51^ are needed.

Additional investigation is needed to identify and isolate the specific source of the ATX-a producing cyanobacteria (although phylogenetic clustering implicates a *Cuspidothrix*/*Aphanizomenon/Dolichospermum/Anabaena and Oscillatoria* sp.) in our samples, and its current and historical distribution in Australia. Although previous studies in Australia^10–13^ have reported the presence of potential ATX-a producers [e.g., *Cu. issatschenkoi* (previously *Aphanizomenon issatschenkoi*)] by microscopy, their toxigenicity was not confirmed. Phylogenetic analyses using 16S rRNA, *rpo*C1 or Phycocyanin intergenic spacer (PC-IGS) marker genes on isolated ATX-a producing strains may provide further insights to the biogeography of ATX-a producers in Australia as has been previously studied for CYN producing *R. raciborskii* (see review by Wilk-Woźniak *et al*.^9^).

## Materials and Methods

### Sample collection and storage

A total of 226 samples representing cyanobacterial blooms were collected between November 2010 to April 2017 from surface water sources across the state of VIC, Australia (Fig. 1). Bloom samples (1 L surface grabs) were collected in polyethylene terephthalate (PETG) bottles. For microscopy, a 50 mL aliquot of the sample was preserved in Lugol’s iodine solution. All samples were dispatched and transported on ice to the laboratory within 24 hours of collection. Upon arrival, 50 mL aliquots of each bloom sample was centrifuged at 3600 × g for 15 min and pellets were stored at −20 °C for genomic DNA extraction and ELISA analysis.

### Microscopy and cyanobacteria identification

Microscopic identification and enumeration of cyanobacteria was carried out on a Nikon ECLIPSE C*i*-H550S light microscope at 200 to 600 times magnification. All taxa were identified to genus and species level using established cyanobacterial taxonomic keys^37,38^. Cell counting was performed on a calibrated Sedgewick Rafter Chamber (Pyser-SGI^®^, United Kingdom) that holds 1 mL of sample over an area of 50 × 20 mm. The biovolume of each taxon was calculated from the cell count data using the “Biovolume calculator”^52^. In circumstances where a taxon was identified to a genus or family level, the mean cell/filament volume of the largest known toxic species within that genus or family was used to calculate the biovolume.

### Genomic DNA extraction and amplification of *ana*C gene by nested PCR

Sample enrichment and genomic DNA extraction was performed using the PowerBiofilm Kit (MoBio, USA) as described elsewhere^53^. Each DNA sample was eluted in 100 µL of 10 mM Tris buffer and stored at −20 °C for PCR analysis. All DNA samples were tested for sample degradation and/or for the presence of PCR inhibitors prior to *ana*C nested PCR assay using cyanobacteria-specific 16S rRNA primers and an internal control as described by Baker *et al*.^53^. Presence of the *ana*C gene (required for ATX-a biosynthesis) was assessed by nested PCR using previously published *ana*C primer pairs (primary PCR: anxgenF/anxgenR and secondary PCR: *ana*C-genF/*ana*C-genR)^22^ (Table 1) with minor modifications to their published protocols. For each step in the nested PCR assay, a 50 µL mastermix consisted of 2.5 mM MgCl_2_, 0.2 mM dNTP, 1X Green GoTaq® Flexi Buffer (Promega, USA), 2.5U of GoTaq® DNA Polymerase (Promega, USA) and 5% dimethyl sulfoxide (DMSO). In the primary PCR, 2 µL genomic DNA and 0.3 µM each primer was added to the mastermix and the following thermocycler protocol was used: an initial denaturation at 94 °C for 2 min followed by 30 cycles of 94 °C for 30 sec (denaturation), 52 °C for 30 sec (annealing), 72 °C for 30 sec (extension) and a final extension step at 72 °C for 5 min.

In the secondary (nested) PCR, 2 µL of PCR product generated from the first PCR reaction was added as the template to the mastermix along with 0.3 µM each of internal primer (i.e., *ana*C-genF and *ana*C-genR). The thermocycling conditions for the secondary PCR were as follows: an initial denaturation at 94 °C for 2 min followed by 30 cycles of 94 °C for 30 sec (denaturation), 58 °C for 30 sec (annealing), 72 °C for 30 sec (extension) and a final extension step at 72 °C for 5 min. All PCR amplifications were run on a BioRad T100™ thermal cycler. A known ATX-a producing *Cu. issatchenkoi* CY1941 was used as a positive control and a known negative ATX-a producer (*Nostoc punctiforme* PCC73102) and non-template control (nuclease free water) were used as negative controls for all PCR reactions [Non-template controls (NTC) from the primary PCR were used as carry-over NTC controls in the secondary PCR]. To determine the size of each amplicon and its specificity, all nested PCR products were subjected to electrophoresis on a 1.5% agarose gel at 90V for 35 min followed by visualisation of bands using a Gel Doc™ XR+ System.

### Sequencing and phylogenetic analysis of *ana*C gene

All nested PCR amplicons were cleaned up prior to sequencing using exoSAP-IT® shrimp alkaline phosphatase. The exoSAP mastermix consisted of Exonuclease I, FastAP™ Thermosensitive Alkaline Phosphatase and 5 µL of nested PCR template. Reactions were incubated in a BioRad T100™ thermal cycler at 37 °C for 30 min followed by 85 °C for 15 min. The purified nested PCR products were then subjected to bi-directional automated sequencing (BigDye® Terminator version 3.1 chemistry, Applied Biosystems, USA) using the internal primers *ana*C-genF and *ana*C-genR. The quality of each sequence was assessed using the program Geneious® Version 10 and sequence identity was confirmed by a BLASTn search of the NCBI (National Centre for Biotechnology Information) non-redundant sequence database. Verified sequences were trimmed and aligned along with published *ana*C sequences from NCBI and adjusted manually using the programs MUSCLE^54^ and Mesquite, respectively^55^. An unrooted Maximum Likelihood (ML) tree based on the Tamura-Nei model^56^ with 1000 bootstrap replicates was constructed using the program MEGA7^57^. The phylogenetic tree was viewed and edited for publication using Adobe Illustrator CC 2017.

### Anatoxin-a analysis using ELISA

This work focused on recently collected (2016–2017) *ana*C*-*positive (n = 32) and representative negative (n = 8) samples identified by nested PCR to reduce the potential for false negative ELISA results due to the physical degradation of the cyanotoxin during storage^58^. Direct testing for total ATX-a (i.e., intracellular and extracellular) toxin was performed in duplicate using the ATX-a ELISA kit (Abraxis^®^, USA) as per the manufacturer’s protocol (available at https://www.abraxiskits.com/wp-content/uploads/2016/06/Anatoxin-a-ELISA-rev053116.pdf). Briefly, samples were freeze/thawed three times to lyse the cells. Prior to testing, the enzyme conjugate and antibody were reconstituted with 3 mL of conjugate and antibody diluent, respectively. Wash buffer (5X) and sample diluent (10X) were diluted at a ratio of 1:5 and 1:10, respectively, using deionised water before use. For each assay, 50 µL of the standard solutions (i.e., standard 0 to standard 5), control and samples were loaded into a 96-well microtitre plate, followed by the addition of 50 µL each of the reconstituted enzyme and antibody solution. After 60 minutes incubation at room temperature, plates were washed four times using 250 µL of 1X diluted wash buffer for each well and each washing step. After drying the plates, 100 µL of substrate colour solution was added to individual wells and contents were incubated for 20–30 minutes at room temperature. Following the addition of stop solution (100 µL), absorbances were read at 450 nm on a Synergy™ hybrid multi-mode microplate reader (BioTek, USA). A standard curve was then constructed by plotting the %B/B_0_ for each of the standards (where B is the mean absorbance value for each of the standards 1 to 5, B_0_ is the mean absorbance reading of standard 0) on the y-axis versus the corresponding anatoxin-a concentration on the x-axis. Finally, %B/B_0_ of each sample (where B is the mean absorbance value for each of the sample, B_0_ is the mean absorbance reading of standard 0) was interpolated on the standard curve to calculate the quantity of total ATX-a in each of the 40 samples. The measurement range for ATX-a ELISA kit stated by manufacturers is from 0.15–5 µg · L^−1^. An ATX-a concentration below 0.15 µg · L^−1^ was considered to be negative or zero µg · L^−1^.

## Statistical analysis

Statistical analyses were computed and displayed using R software^59^. Welch’s two sample *t*-test was used to compare log-transformed ATX-a ELISA concentrations between nested PCR-positive and negative samples. Further, the relationship between nested PCR band intensities and log-transformed ELISA concentrations was compared using ANOVA followed with Tukey’s honest significant difference (HSD) test. Significance was accepted for *p* < 0.05.

## Supplementary information

Supplementary dataset 1

## Data Availability

A subset of nucleotide sequences representing *ana*C sequences derived from this study are available from NCBI GenBank (https://www.ncbi.nlm.nih.gov/nucleotide) under Accession Numbers MG836989 - MG836992. Additional Table S1 attached as Supplementary Information.
