# Supplementary material for: First report of anatoxin-a producing cyanobacteria in Australia illustrates need to regularly up-date monitoring strategies in a shifting global distribution
Source: Sci Rep. 2019 Jul 26;9:10894. doi: 10.1038/s41598-019-46945-8 (PMC6659621; doi:10.1038/s41598-019-46945-8)
Supplement: Supplementary file 1 — Supplementary dataset 1 [file 41598_2019_46945_MOESM1_ESM.pdf]

First report of anatoxin-a producing cyanobacteria in Australia illustrates need to regularly up-date monitoring strategies in a shifting global distribution.

Nijoy John [1, 2, *], Louise Baker [2], Brendan R.E. Ansell [2], Steven Newham [3], Nicholas D. Crosbie [4, 5], Aaron R. Jex [1, 2]

[1] *Faculty of Veterinary Science, The University of Melbourne, Parkville, Victoria 3010, Australia*

[2] *Population Health and Immunity Division, Walter and Eliza Hall Institute of Medical Research, Parkville, Victoria 3052, Australia*

[3] *Goulburn Valley Water, Shepparton, Victoria 3630, Australia*

[4] *Melbourne Water, Docklands, Victoria 3008, Australia*

[5] *School of Civil and Environmental Engineering, The University of New South Wales, Sydney, NSW, 2052, Australia*

* Corresponding author. Current address: Population Health and Immunity Division, Walter and Eliza Hall Institute of Medical Research, Parkville, Victoria 3052, Australia. Tel.: +61 425490792; E-mail address: njohn@student.unimelb.edu.au; john.n@wehi.edu.au (Nijoy John

Table S1. Microscopy, nested PCR, sequencing and ELISA results of a subset of cyanobacterial bloom samples tested in this study. Symbols; +, faint PCR band; ++, strong PCR band; +++, very strong PCR band; NA, not analysed. Previously reported ATX-a producing cyanobacteria characterised by isolation studies, molecular analysis and/or found to carry ATX-a synthetase gene required for ATX-a production are shown in bold. [a]; cell numbers (cells · mL^{-1})/biovolume (mm^3 · L^{-1}) of potential ATX-a producers shown in bold.

Sample number	Year sample collected	Location	PCR result	NCBI BLASTn (% identity to *ana*C gene)	ELISA (µg · L^{-1})	Microscopy[a]
BGAUM1	2016	Yarrawonga	+	NA	NA	*Chrysosporum ovalisporum, Cyanogranis, Planktolyngbya, Aphanocapsa,* ***Cuspidothrix issatschenkoi*** **(736/0.04)**
BGAUM2	2016	Yarrawonga	+	NA	NA	*C. ovalisporum, Cyanogranis, Planktolyngbya, Snowella, Aphanocapsa, Pseudanabaena,* ***C. issatschenkoi*** **(326/0.01)*,*** ***Dolichospermum*** **sp. (117/0.03)**
BGAUM3	2016	Yarrawonga	+	NA	NA	*C. ovalisporum, Planktolyngbya, Snowella, Cyanogranis*
BGAUM4	2016	Yarrawonga	++	KX096813.1 Uncultured cyanobacterium (100%), JF803655.1 *Aphanizomenon* sp. 3 (99.7%), KM245024.1 *Cuspidothrix issatschenkoi* (99.4%), JF803647.1 *Dolichospermum circinale* 86 (89.7%)	0.2	*C. ovalisporum, Planktolyngbya, Cyanogranis, Aphanocapsa, Pseudanabaena*
BGAUM5	2016	Yarrawonga	+	NA	0.2	*C. ovalisporum, Cyanogranis, Aphanocapsa, Planktolyngbya, Cyanodictyon,* ***Dolichospermum*** **sp. (447/0.19)**
BGAUM8	2016	Wahgunyah	+	NA	0.15-0.2	*C. ovalisporum, Planktolyngbya, Merismopedia,* ***Dolichospermum*** **sp. (512/0.14)**
BGAUM9	2016	Wahgunyah	+	NA	NA	*C. ovalisporum, Planktolyngbya, Aphanocapsa, Cyanogranis*
BGAUM11	2016	Mulwala	+	NA	0.2	*C. ovalisporum, Aphanocapsa, Planktolyngbya,* ***Dolichospermum*** **sp. (75/0.02)*,*** ***C. issatschenkoi*** **(102/0.005)**
BGAUM14	2016	MV channel	+++	KP036898.1 Uncultured cyanobacterium (100%),	0.3	*C. ovalisporum, Planktolyngbya, Aphanocapsa, Cyanogranis,*

				KM245024.1 *C. issatschenkoi* (99.7%), JF803655.1 *Aphanizomenon* sp.3 (99.4%), JF803647.1 *D. circinale* 86 (89.7%)		
BGAUM16	2016	MV channel	+	NA	0.15-0.2	*C. ovalisporum, Planktolyngbya, Aphanocapsa, Cyanogranis,* ***Dolichospermum*** **sp. (80/0.02)**
BGAUM17	2016	Picola	+	NA	<0.15	*C. ovalisporum, Planktolyngbya, Chroococcales, Geltierinema,* ***Dolichospermum*** **sp. (220/0.078)**
BGAUM19	2016	Numurkah	+	NA	0.2	*C. ovalisporum, Planktolyngbya, Chroococcales, Raphidiopsis raciborskii*
BGAUM23	2016	Barmah	+	NA	NA	*C.ovalisporum, Chroococcales, Planktolyngbya, C. raciborskii,* ***C. issatschenkoi*** **(116/0.006)**
BGAUM25	2016	Cobram	++	KX096813.1 Uncultured cyanobacterium (100%), JF803655.1 *Aphanizomenon* sp. 3 (99.7%), KM245024.1 *Cuspidothrix issatschenkoi* (99.4%), JF803647.1 *Dolichospermum circinale* 86 (89.7%)	NA	*C. ovalisporum, Chroococcales, Planktolyngbya,*
BGAUM27	2016	Numurkah	+	NA	0.8	*C. ovalisporum, Chroococcales, Planktolyngbya, R. raciborskii*
BGAUM28	2016	Numurkah	+	NA	NA	*C. ovalisporum, Chroococcales, Planktolyngbya, R. raciborskii*
BGAUM29	2016	Nathalia	+	NA	NA	*C. ovalisporum, Chroococcales, Planktolyngbya,*
BGAUM30	2016	Barmah	+	NA	0.15-0.2	*C. ovalisporum, Chroococcales, Planktolyngbya,* ***C. issatschenkoi*** **(77/0.004),** ***Dolichospermum*** **sp. (115/0.032)**
BGAUM32	2016	Gunbower	+	NA	NA	*C. ovalisporum, Planktolyngbya*
BGAUM33	2016	Echuca	+	NA	NA	*C. ovalisporum, Cyanodictyon, Cyanogranis, Planktolyngbya, Aphanocapsa, Snowella*
BGAUMLB33	2011	Merrimu	+	NA	NA	NA
BGAUM35	2016	Pental	+	NA	NA	*C.ovalisporum, Aphanocapsa, Planktolyngbya*
BGAUM38	2016	Torrumbarry	+++	KX096813.1 Uncultured cyanobacterium (100%), JF803655.1 *Aphanizomenon* sp. 3 (99.7%), KM245024.1 *Cuspidothrix issatschenkoi* (99.4%), JF803647.1	NA	*C. ovalisporum, Planktolyngbya, Aphanocapsa,* ***C. issatschenkoi*****, (77/0.004)** *Aphanothece, R. raciborskii*

				Dolichospermum circinale 86 (89.7%)		
BGAUMLB39	2011	Merrimu	+	NA	NA	NA
BGAUMLB40	2011	Pykes Creek	+	NA	NA	NA
BGAUM45	2016	Piangil	+	NA	NA	*C. ovalisporum, Planktolyngbya, Aphanocapsa, Cyanogranis, Microcystis,*
BGAUM46	2016	Nyah	+++	KP036898.1 Uncultured cyanobacterium (100%), KM245024.1 *C. issatschenkoi* (99.7%), JF803655.1 *Aphanizomenon* sp.3 (99.4%), JF803647.1 *D. circinale* 86 (89.7%)	0.5	*C. ovalisporum,* ***C. issatschenkoi*** **(82/0.004),** *Planktolyngbya, Cyanogranis, Cyanodictyon, Aphanocapsa*
BGAUM48	2016	Nyah	+++	KP036898.1 Uncultured cyanobacterium (100%), KM245024.1 *C. issatschenkoi* (99.7%), JF803655.1 *Aphanizomenon* sp.3 (99.4%), JF803647.1 *D. circinale* 86 (89.7%)	0.2	*C. ovalisporum, Planktolyngbya, Aphanocapsa, Cyanogranis,* ***C. issatschenkoi*** **(112/0.006)**
BGAUM52	2016	Pental	+	NA	NA	*C. ovalisporum, Plantolyngbya, Cyanodictyon, Aphanotece*
BGAUM56	2016	Yarrawonga	+	NA	NA	*C.ovalisporum, Planktolyngbya, Cyanogranis****,*** ***Dolichospermum*** **sp. (200/0.056)**, ***C. issatschenkoi*** **(99/0.005)**
BGAUM59	2016	Torrumbarry	+	NA	NA	*C. ovalisporum, Planktolyngbya, Aphanocapsa, Cyanogranis, Aphanothece, C. raciborskii &* ***Dolichospermum*** **sp. (83/0.02)**
BGAUM67	2016	Wemen	+	NA	0.3	*Aphanocapsa, Aphanothece, Cyanogranis, C. ovalisporum,* ***Dolichospermum*** **sp. (1629/0.7)*****, C. issatschenkoi*** **(106/0.006)**
BGAUM77	2016	Wemen	+	NA	NA	*Aphanocapsa, Aphanothece, Cyanodicyon, Cyanogranis, Planktolyngbya, C. ovalisporum,* ***Dolichospermum*** **sp. (177/0.05)**, ***C. issatschenkoi*** **(265/0.01)**
BGAUM82	2016	Piangil	+++	KP036898.1 Uncultured cyanobacterium (100%), KM245024.1 *C. issatschenkoi* (99.7%), JF803655.1 *Aphanizomenon* sp.3 (99.4%),	0.5	*C. ovalisporum, Planktolyngbya, Aphanocapsa,* ***C. issatschenkoi*** **(1129/0.06)**, *Pseudanabaena, Cyanogranis*

				JF803647.1 *D. circinale* 86 (89.7%)		
BGAUM86	2016	Pental	+++	KP036898.1 Uncultured cyanobacterium (100%), KM245024.1 *C. issatschenkoi* (99.7%), JF803655.1 *Aphanizomenon* sp.3 (99.4%), JF803647.1 *D. circinale* 86 (89.7%)	0.5	*C. ovalisporum, Planktolyngbya, Cyanogranis, Aphanocapsa,* ***C. issatschenkoi*** **(93/0.005),** ***Dolichospermum*** **sp. (54/0.023),**
BGAUM88	2016	Piangil	+++	KP036898.1 Uncultured cyanobacterium (100%), KM245024.1 *C. issatschenkoi* (99.7%), JF803655.1 *Aphanizomenon* sp.3 (99.4%), JF803647.1 *D. circinale* 86 (89.7%)	0.5	*C. ovalisporum, Planktolyngbya, Cyanogranis,* ***C. issatschenkoi*** **(581/0.03)**
BGAUM89	2016	Wemen	+	NA	NA	*Aphanocapsa, Planktolyngbya, Cyanogranis*
BGAUM92	2016	Mulwala	+	NA	NA	*C. ovalisporum, Planktolyngbya, cyanogranis,*
BGAUM93	2016	Nyah	+++	KP036898.1 Uncultured cyanobacterium (100%), KM245024.1 *C. issatschenkoi* (99.7%), JF803655.1 *Aphanizomenon* sp.3 (99.4%), JF803647.1 *D. circinale* 86 (89.7%)	0.35	*C. ovalisporum, Planktolyngbya, Aphanocapsa, Cyanogranis,* ***C. issatschenkoi*** **(2060/0.117)**, *Pseudanabaena, Aphanothece,*
BGAUM94	2016	Numurkah	+	NA	NA	*C. ovalisporum, Planktolyngbya, Chroococcales, R. raciborskii*
BGAUM96	2016	Nathalia	+++	KP036898.1 Uncultured cyanobacterium (100%), KM245024.1 *C. issatschenkoi* (99.7%), JF803655.1 *Aphanizomenon* sp.3 (99.4%), JF803647.1 *D. circinale* 86 (89.7%)	1.6	*C. ovalisporum, Chroococcales, Planktolyngbya,*
BGAUM98	2016	Numurkah	+++	KX096813.1 Uncultured cyanobacterium (100%), JF803655.1 *Aphanizomenon* sp. 3 (99.7%), KM245024.1 *Cuspidothrix issatschenkoi* (99.4%), JF803647.1	NA	*C. ovalisporum, Planktolyngbya, Chroococcales, R. raciborskii*

				Dolichospermum circinale 86 (89.7%)		
BGAUM99	2016	Barmah	+	NA	NA	*C. ovalisporum, Chroococcales, Planktolyngbya, R. raciborskii,* ***C. issatschenkoi*** **(116/0.006)**
BGAUM102	2016	Leitchville	+	NA	NA	*C. ovalisporum, Planktolyngbya, Chroococcales*
BGAUM105	2016	Pental	+++	KP036898.1 Uncultured cyanobacterium (100%), KM245024.1 *C. issatschenkoi* (99.7%), JF803655.1 *Aphanizomenon* sp.3 (99.4%), JF803647.1 *D. circinale* 86 (89.7%)	0.2	*Planktolyngbya, Aphanocapsa, Cyanogranis, Pseaudanabaena, C. ovalisporum, R. raciborskii*
BGAUM109	2016	Yarrawonga	+++	KP036898.1 Uncultured cyanobacterium (100%), KM245024.1 *C. issatschenkoi* (99.7%), JF803655.1 *Aphanizomenon* sp.3 (99.4%), JF803647.1 *D. circinale* 86 (89.7%)	0.2	*C. ovalisporum, Planktolyngbya, Cyanogranis, Cyanodictyon,* ***C. issatschenkoi*** **(588/0.03)**, ***Dolichospermum*** **sp. (100/0.007)** *Microcystis*
BGAUM110	2016	Yarrawonga	n	NA	<0.15	*C. ovalisporum, Cyanogranis, Planktolyngbya, Pseudanabaena, Aphanothece,* ***C. issatschenkoi*** **(114/0.006)*****, Dolichospermum*** **sp. (64/0.001).**
BGAUM135	2016	Pental	n	NA	NA	NA
BGAUM156	2016	Pental	n	NA	<0.15	*C. ovalisporum, Chroococcales, Planktolyngbya,* ***C. issatschenkoi*** **(4073/0.23),** ***Dolichospermum*** **sp. (940/0.08)**
BGAUM160	2016	Yarrawonga	n	NA	<0.15	*C.ovalisporum, Cyanogranis,Planktolyngbya*
BGAUM165	2016	Wemen	n	NA	NA	*C. ovalisporum, Planktolyngbya, Aphanocapsa, Cyanogranis*
BGAUM171	2016	Nyah	n	NA	NA	*Planktolyngbya, Cyanogranis, Cyanodictyon, Aphanocapsa*
BGAUM175	2016	Wemen	n	NA	NA	*C. ovalisporum, Chroococcales, Planktolyngbya,* ***C. issatschenkoi*** **(24/0.001)**
BGAUMLB176	2012	Rowville	+	NA	NA	NA
BGAUMLB177	2012	Monbulk	+	NA	NA	NA
BGAUMLB179	2011	Greaves Road	+	NA	NA	NA

BGAUMLB180	2011	Greaves Promenade	+	NA	NA	NA
BGAUM186	2016	Yarrawonga	n	NA	NA	*C. ovalisporum, Planktolyngbya, Cyanogranis, Aphanocapsa,*
BGAUM187	2016	Yarrawonga	n	NA	NA	*C. ovalisporum, Planktolyngbya, Cyanogranis*
BGAUMLB189	2011	Derrinal	+	NA	NA	NA
BGAUM190	2016	Yarrawonga	n	NA	NA	*C. ovalisporum, Planktolyngbya, Aphanocapsa, Cyanogranis,* ***Dolichospermum*** **sp. (84/0.02)** *and Microcystis*
BGAUM194	2016	Mulwala	n	NA	NA	*C. ovalisporum, Cyanogranis, Planktolyngbya*
BGAUM196	2016	Echuca	n	NA	NA	*C. ovalisporum, Planktolyngbya, Cyanogranis*
BGAUMLB201	2011	Greaves Road	+	NA	NA	NA
BGAUM202	2016	Cobram	n	NA	NA	*C. ovalisporum, Chroococcales, Planktolyngbya, Pseudanabaena*
BGAUM206	2016	Barmah	n	NA	NA	*C. ovalisporum, Planktolyngbya, Chroococcales,* ***C. issatschenkoi*** **(525/0.02),** ***Dolichospermum sp.*** **(99/0.007)**
BGAUM211	2016	Wemen	+++	KP036898.1 Uncultured cyanobacterium (100%), KM245024.1 *C. issatschenkoi* (99.7%), JF803655.1 *Aphanizomenon* sp.3 (99.4%), JF803647.1 *D. circinale* 86 (89.7%)	0.4	*C. ovalisporum, Chroococcales, Planktolyngbya, Microcystis,* ***C. issatschenkoi*** **(7/0.0004)**
BGAUM212	2016	Wemen	+	NA	NA	*C. ovalisporum, Chroococcales, Planktolyngbya,* ***C. issatschenkoi*** **(20/0.001),** ***Dolichospermum*** **sp. (148/0.063)**
BGAUM216	2016	Pental	+	NA	0.3	*C. ovalisporum, Planktolyngbya, Chroococcales*
BGAUM217	2016	Barmah	n	NA	NA	*C. ovalisporum, Chroococcales, Planktolyngbya,* ***C. issatschenkoi*** **(539/0.03),** ***Dolichospermum*** **sp. (299/0.04)**
BGAUM225	2016	Piangil	+	NA	0.4	*C. ovalisporum, Planktolyngbya, Chroococcales,* ***C. issatschenkoi*** **(542/0.03)**
BGAUM239	2016	Nyah	n	NA	NA	NA
BGAUM254	2016	Pental	+++	KP036898.1 Uncultured cyanobacterium (100%), KM245024.1 *C. issatschenkoi*	0.7	*C. ovalisporum, Chroococcales, Planktolyngbya*

				(99.7%), JF803655.1 *Aphanizomenon* sp.3 (99.4%), JF803647.1 *D. circinale* 86 (89.7%)		
BGAUM263	2016	Nyah	+++	KP036898.1 Uncultured cyanobacterium (100%), KM245024.1 *C. issatschenkoi* (99.7%), JF803655.1 *Aphanizomenon* sp.3 (99.4%), JF803647.1 *D. circinale* 86 (89.7%)	0.5	*C. ovalisporum, Planktolyngbya, Chroococcales*
BGAUM269	2016	Wemen	+	NA	0.3	*C. ovalisporum, Microcystis, Chroococcales,* ***C. issatschenkoi*** **(43/0.002),** ***Dolichospermum*** **sp. (67/0.02)**
BGAUM299	2016	Torrumbarry	+	NA	NA	
BGAUM301	2016	Piangil	n	NA	<0.15	*C. ovalisporum, Planktolyngbya, Chroococcales*
BGAUM313	2016	Mulwala	+	NA	NA	*C. ovalisporum, Aphanocapsa, Chroococcales*
BGAUM324	2016	Leitchville	n	NA	NA	NA
BGAUM372	2016	Nyah	n	NA	0.3	*C. ovalisporum, Planktolyngbya, Chroococcales*
BGAUM376	2016	Pental	+++	KP036898.1 Uncultured cyanobacterium (100%), KM245024.1 *C. issatschenkoi* (99.7%), JF803655.1 *Aphanizomenon* sp.3 (99.4%), JF803647.1 *D. circinale* 86 (89.7%)	0.8	*C. ovalisporum, Chroococcales, Planktolyngbya*
BGAUM700	2017	Picola	+++	JF803653.1 *Oscillatoria* sp. PCC 9240 (100%), %), KX016036 *P. autumnale* CAWBG618 (100%)	>4	*C. ovalisporum, Aphanocapsa, Planktolyngbya*
BGAUM701	2017	Picola	+++	NA	NA	*C. ovalisporum, Aphanocapsa, Planktolyngbya*
BGAUM702	2017	Goulburn Weir	+++	Poor quality sequence	1	*Chrococcales, Planktolyngbya, C. ovalisporum, Romeria*
BGAUM704	2017	Kirwans bridge	+++	KT246302.1 Uncultured cyanobacterium (94.7%), JF803647.1 *D. circinale* 86 (94.7%), JF803655.1	>4	*C. ovalisporum, Chrococcales, Planktolyngbya,* ***Dolichospermum*** **sp.**

				Aphanizomenon sp.3, KM245024.1 *C. issatschenkoi* (90%)		
BGAUM709	2017	Boonie Doon	++	JF803653.1 *Oscillatoria* sp. PCC 9240 (100%), %), KX016036 *P. autumnale* CAWBG618 (100%)	1.5	*Planktolyngbya, Pseaudanabaena, Aphanocapsa, Merismopidia,* ***Phormidium* (1178/0.11),** *Cyanogranis, Microcystis*
BGAUM710	2017	Numurkah	++	KP036898.1 Uncultured cyanobacterium (100%), KM245024.1 *C. issatschenkoi* (99.7%), JF803655.1 *Aphanizomenon* sp.3 (99.4%), JF803647.1 *D. circinale* 86 (89.7%)	0.2	***Dolichospermum* sp. (4,754,287/1331.2)**, *Microcystis, Planktolyngbya, Pseaudanabaena,*
BGAUM711	2017	Waranga	n	NA	<0.15	*C. ovalisporum, Aphanothece, Aphanocapsa,* ***D. circinale* (7827/1.95)**, *Cyanogranis, Cyanodictyon, Planktolyngbya*
BGAUM714	2017	Katandra	n	NA	<0.15	*Chrococcales, C. ovalisporum, Planktolyngbya,* ***Phormidium* (8947/1.07),** *Leptolyngbya, Pseudanabena*
BGAUM716	2017	Rushworth	n	NA	<0.15	*C. ovalisporum, Chrococcales, Snowella, Psuedanabaena,*
BGAUM720	2017	Numurkah	+++	KT246302.1 Uncultured cyanobacterium (94.7%), JF803647.1 *D. circinale* 86 (94.7%), JF803655.1 *Aphanizomenon* sp.3, KM245024.1 *C. issatschenkoi* (90%)	NA	*Chrococcales, Planktolyngbya,* ***Dolichospermum* sp. (1042/0.17)**

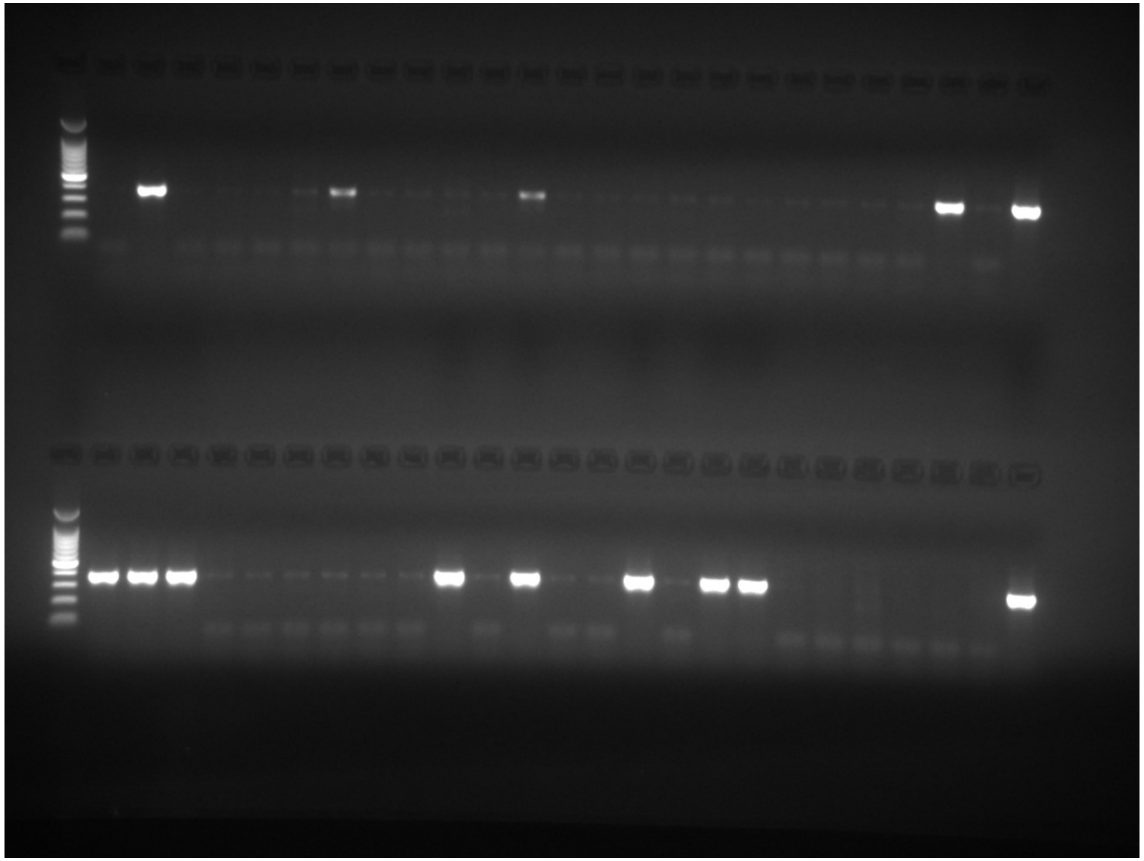

Figure S1. Uncropped, full-length gel image of the nested PCR results of anatoxin-a synthetase C (*ana*C) gene of a subset of cyanobacterial bloom samples separated by 1.5 % agarose gel electrophoresis.
